# Lithium nitrate salt-assisted CO_2_ absorption for the formation of corrosion barrier layer on AZ91D magnesium alloy†

**DOI:** 10.1039/d4ra02829e

**Published:** 2024-06-03

**Authors:** Gyoung G. Jang, Jiheon Jun, Jong K. Keum, Yi-Feng Su, Mayur Pole, Sridhar Niverty, Vineet V. Joshi

**Affiliations:** a Manufacturing Science Division, Oak Ridge National Laboratory (ORNL) Oak Ridge TN 37831 USA jangg@ornl.gov; b Materials Science and Technology Division, ORNL USA junj@ornl.gov suy1@ornl.gov; c Center for Nanophase Materials Science and Neutron Scattering Division, ORNL USA keumjk@ornl.gov; d Energy and Environment Directorate, Pacific Northwest National Laboratory (PNNL) Richland WA 99354 USA mayur.pole@pnnl.gov sridhar.niverty@pnnl.gov vineet.joshi@pnnl.gov

## Abstract

Mg alloy corrosion susceptibility is a major issue that limits its wide industrial application in transport, energy and medical sectors. A corrosion-resistant layer containing crystalline MgCO_3_ was formed on the surface of AZ91D Mg alloy by Li salt loading and thermal CO_2_ treatment. Compared to the uncoated AZ91D surface, the surface layer exhibited up to a ∼15-fold increase in corrosion resistance according to the electrochemical results in 3.5 wt% NaCl solution and ∼32% decrease in wear rate compared to untreated AZ91D. The improved corrosion resistance is attributed to the formation of a <10 μm thick dense layer containing Mg, O, C and Li with crystalline MgCO_3_ phases. The initial step was to form a porous MgO layer on the surface of AZ91D Mg alloy, followed by loading an alkali metal salt (*i.e.*, LiNO_3_) onto the MgO surface. The porous MgO surface was then reconstructed into a dense insulation layer containing Mg carbonate through CO_2_ absorption facilitated by molten Li salt during thermal CO_2_ treatment at 350 °C. As a potential method to utilize excessive CO_2_ for beneficial outcomes, the formation of the carbonate-containing film introduced in this study opens a new pathway for protecting various existing Mg alloys for diverse industrial applications.

## Abstract 2

The formation of Mg–Li–C–O barrier layer on AZ91D Mg alloy using LiNO_3_-assisted thermal CO_2_ treatment provides a pathway to utilize excessive CO_2_ for corrosion protection of AZ91D.

## Graphical abstract

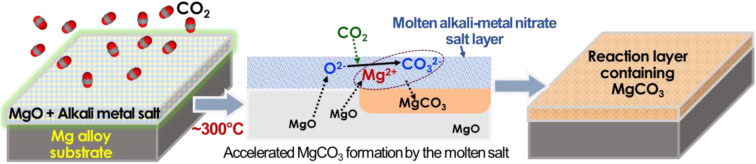

## Introduction

Magnesium (Mg) alloys are among the lightest structural materials and have a strong potential for constructing lightweight engineered systems. The use of such lightweight materials would reduce energy consumption and carbon emission, particularly in high-volume transportation applications.^1–3^ The other emerging applications of Mg alloys are in bioengineering implants and battery and fuel cell electrodes.^4–7^ Despite the long history of Mg alloy development, the widespread use of Mg alloys has been restricted by several intrinsic chemical and mechanical limitations including corrosion susceptibility,^1,8–12^ poor formability,^2,13^ and low creep resistance.^14^ Recent development efforts for new Mg alloy compositions (*e.g.*, rare earth alloying) enabled overcoming these physical and mechanical material drawbacks.^15,16^ However, in most cases, these alloying strategies for mechanical property enhancement, such as creep resistance and/or strength, did not improve or even decrease corrosion resistance.^17^ Conversely, alloying for corrosion resistance, such as Ca doping, did not show mechanical property enhancement.^18,19^ Still, corrosion of Mg alloys is the most critical issue that needs to be addressed.

Protective surface coatings are still considered the most cost-effective way to improving the corrosion resistance of Mg alloys without compromising the beneficial mechanical properties obtained through alloying. Various coating approaches have been examined for Mg and Mg alloys, including superhydrophobic coatings,^20–22^ plasma electrolytic oxidation and anodization,^23–26^ inorganic chemical and fluoride coatings,^27–30^ cold spray coatings^31–34^ or polymer coatings.^35,36^ However, a single coating alone is insufficient to provide adequate protection for Mg surfaces in automotive body applications. As a result, multilayer coating schemes using wet chemical processes have been proposed to ensure reliable Mg protection.^1,37^ It is important to consider the standard regulation set by the US Environmental Protection Agency since these wet-chemical-based coatings can generate volatile organic compounds. These conditions cause complexities in the processes, increase costs, and may result in insufficient corrosion protection.

Magnesium carbonate has the potential to serve as a corrosion barrier material. It is a stable compound with a very low solubility of 14 mg per 100 mL in water. This compound can remain inert in water for extended periods and thermodynamically favor the growth of a layer on the MgO surface in air.^38,39^ Although the formation of MgCO_3_ on the MgO surface (MgO(s) + CO_2_(g) ↔ MgCO_3_(s), Δ*G* = −21.4 kJ mol^−1^) is a spontaneous and thermodynamically favorable reaction,^40,41^ the growth of the carbonate layer is limited to the surface due to slow reaction kinetics. As a result, only a few nanometer-thick layers were formed as reported previously.^42^ Such thin layers cannot provide sufficient corrosion protection for bulk samples. To accelerate the growth of the MgCO_3_ film, Wang and others^40^ utilized excited CO_2_ to form a MgCO_3_ layer on pure Mg nanorods under electron-beam irradiation, while Jang and others^43^ employed atmospheric CO_2_ plasma to create a ∼500 nm thick carbon-rich layer on pure Mg, resulting in improved corrosion resistance on a larger scale. However, the achievement of a protective layer with sufficient coverage on Mg alloys has not been demonstrated.^41^

Recently, MgO-based mesoporous structures have received significant attention for energy-efficient CO_2_ capture. These low-cost materials exhibit a high theoretical capture capacity (*e.g.*, 1.09 g CO_2_ per 1 g MgO) and demonstrate a wide sorption temperature range (25–350 °C).^44^ However, the limited mass transfer in bulk solid sorbents, such as micro MgO sorbent particles, poses a key technical challenge for CO_2_ absorption, resulting in only 5–20% sorption capacity even at room temperature and even high temperature (*e.g.*, 200 °C). To address this technical issue, alkali nitrate molten salts can be employed to coat the MgO surface. The molten salt coating significantly enhances the CO_2_ sorption capacity, reaching up to ∼87% at intermediate temperature, thereby enabling a high yield of MgCO_3_.^45^ The molten salts, such as NaNO_3_, NaNO_2_, LiNO_3,_ and KNO_3_, act as reaction media, dissolving both CO_2_ and MgO, thereby facilitating the kinetics of the carbonate formation reaction.^46,47^

**Fig. 1:**
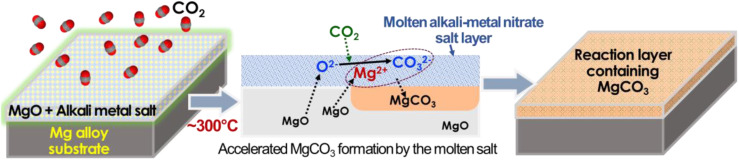
Schematic description of thermal CO_2_ treatment on Mg alloy specimens to form the corrosion barrier layer. The middle schematic is a graphical description of a key MgCO_3_ formation mechanism reported in ref. 48.

Inspired by the concept of molten salt coating on MgO particles for high-capacity CO_2_ absorption, we have explored a novel approach to form a dense and thick carbonate layer on the surface of Mg alloy metal, serving as an effective corrosion barrier. The approach involves enhancing the formation of a MgCO_3_ layer on a pre-formed MgO surface through thermal treatment of the salt-coated MgO surface in a CO_2_-rich environment, as schematically described in Fig. 1. Initially, the Mg alloy surface was treated with an aqueous salt solution, resulting in the formation of a porous and cracked MgO surface layer. Subsequently, an aqueous LiNO_3_ solution was coated on the pre-formed MgO surface and dried to form a solid salt layer. The CO_2_ thermal treatment, conducted at an intermediate temperature (300–350 °C), above the melting point of LiNO_3_ (253 °C), facilitated CO_2_ absorption through the molten salt layer, effectively converting the MgO layer to MgCO_3_.

The interaction of MgO with CO_2_ in molten LiNO_3_ supposedly occurs as follows (as described in Fig. 1):^1^ oxygen ion (O^2−^) and CO_2_ reaction forming CO_3_^2−^ in the molten salt layer and^2^ chemical reaction between CO_3_^2−^ and Mg^2+^ forming MgCO_3_. While the key mechanism of molten salt-assisted MgCO_3_ formation has been described in previous literature,^45,46,49^ those studies focused on zero-dimensional nano/microparticles for high-capacity CO_2_ absorption rather than large-scale 2D surfaces for corrosion mitigation. To the best of our knowledge, no study has proposed the utilization of this MgCO_3_ formation reaction to form a corrosion barrier coating for Mg alloys.

In this study, we conducted a proof-of-principle evaluation of the formation of a corrosion barrier layer with Mg–O–C–Li components. Various corrosion tests were performed, and advanced electron microscopic/X-ray-based chemical analyses were utilized to characterize the presence of carbon and Li. Additionally, real-time XRD analysis was employed to understand the formation mechanism. By simply using CO_2_ as the feeding gas, this approach has the potential to provide effective corrosion protection for various complex Mg alloy structures, including inner tube surfaces, 3D structures and other cast Mg structures. The tribological properties were also elucidated in this study to understand the role of the barrier layer formed by CO_2_ thermal treatment for improved surface wear properties. Moreover, the CO_2_ thermal treatment technique offers the advantage of minimizing environmental concerns associated with waste treatments often required by traditional wet-chemical-based methods for protective coatings on Mg alloys.

## Experimental procedure

### Materials

A commercial high-pressure die-cast AZ91D was obtained and analyzed for the chemical composition, which is summarized in Table 1. Test samples of AZ91D with the dimensions of 25 mm × 25 mm and thickness of ∼2 mm were prepared by electric discharge machining. The test samples were wet ground with silicon carbide (SiC) paper to a 600-grit finish, followed by ultrasonic cleaning in deionized water and drying with compressed air. Prior to CO_2_ thermal treatment, the samples were stored in a desiccator. All salt chemicals were purchased from Sigma-Aldrich, St. Louis, MO, USA.

**Table 1:** Chemical composition of high-pressure die-cast AZ91D Mg alloy in wt%, analyzed by ICP-OES technique^50^

Mg	Al	Zn	Mn	Minor elements: Cu, Fe & Ni
89.95	9.24	0.59	0.22	<0.001

### Preparation of Li salt CO_2_-thermal-treated AZ91D samples

First, 600-grit SiC finished AZ91D coupons (25 × 25 mm^2^) were immersed in a 2.5 wt% aqueous NaHCO_3_ solution for 10 min to form a MgO layer on the surface. 10 or 25 wt% LiNO_3_ solution was prepared. An assigned amount of Li solution (0.1 mL) was drop-coated onto the pre-formed MgO surface, and the treated samples were dried in a convection oven at 60 °C. For example, a sample designated as 2.5–2.5 indicates that it was prepared by immersing the sample in a 2.5 wt% NaHCO_3_ solution for 10 min to form MgO, followed by loading it with 0.1 mL of a 25 wt% LiNO_3_ solution. Table 2 summarizes the sample nomenclatures and preparation conditions used for AZ91D.

**Table 2:** Details of surface treatment preparation

Sample nomenclature	NaHCO_3_ concentration in wt%	Immersion time (min)	LiNO_3_ loading (wt% in 0.1 mL)	Treatment gas
MgO	2.5	10	0	CO_2_
Li-0.5-1.0	0.5	10	10	CO_2_
Li-2.5-1.0	2.5	10	10	CO_2_
Li-2.5-2.5	2.5	10	25	CO_2_
Li-2.5-2.5 w/Ar	2.5	10	25	Ar
Li-2.5-5.0	2.5	10	25 + 25^a^	CO_2_

a LiNO_3_ loading occurred twice.

**Fig. 2:**
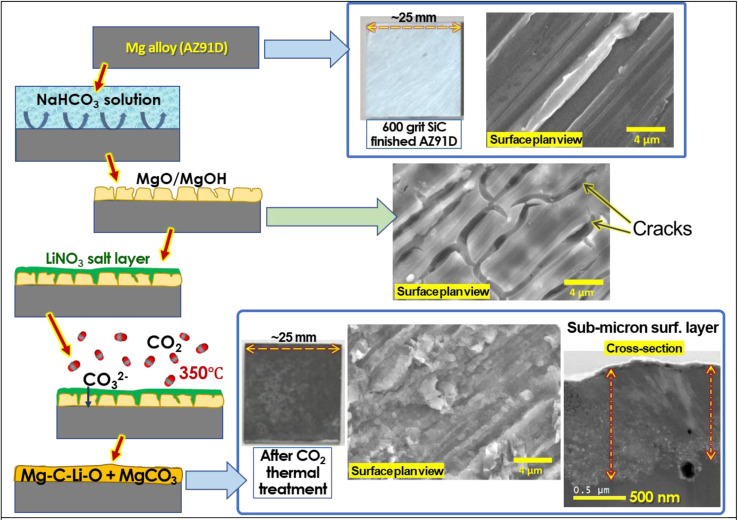
Schematic of Li salt thermal CO_2_ treatment on Mg alloy specimens to form a corrosion protective layer with optical and microscopic images. Pristine AZ91D Mg alloy was treated to form corrosion products (*i.e.*, MgO and MgOH_2_) with immersion in an aqueous NaHCO_3_ 2.5 wt% solution. The surface with porous MgO/MgOH_2_ was covered by 0.1 mL of 25 wt% LiNO_3_. Next, thermal CO_2_ annealing at 350 °C was performed to form the Mg–C–Li–O + MgCO_3_ layer by the reactions of CO_2_, MgO and molten LiNO_3_. The surface and cross-sectional sample images at the bottom right were from a Li-0.5-1.0 sample.

Thermal CO_2_ treatment was conducted using a 3-zone controlled alumina tube furnace (*i.e.*, 1370 mm in length, 82 mm in diameter). The furnace temperature was raised to 350 °C over 3 h with argon purging gas. Once the target temperature was reached, lab-grade clean CO_2_ (99.995% purity) was supplied to the furnace at a flow rate of 300 mL min^−1^ for 3 h. After completing the treatment, both the furnace and CO_2_ supply were shut off, and argon purging was performed until the samples were cooled to 50 °C or below. The treated AZ91D samples were washed with deionized water under sonication for 5 min and then dried for further analysis. A graphic description of the entire treatment process can be found in Fig. 2.

### Characterization of materials

Surface-treated AZ91D samples were analyzed by scanning electron microscopy (SEM) equipped with energy-dispersive X-ray spectroscopy (EDS), X-ray diffraction (XRD) and scanning transmission electron microscopy (STEM). The specimens for STEM analysis were prepared *via* the focus ion beam (FIB) milling technique by the *in situ* lift-out method using a Hitachi NB5000 dual-beam FIB-SEM. Prior to milling, protective layers of tungsten and carbon (4 mm wide, 20 mm long, and 0.5 mm thick) were deposited on the sample to preserve the area of interest during ion sputtering. Higher resolution microstructure image and elemental mapping in cross-section were carried out using a FEI model Talos F200X STEM operated at 200 kV, which is equipped with an extreme field emission gun (X-FEG) electron source and Super-X EDS (energy dispersive spectroscopy) system with 4 silicon drift detectors (SDD) and a JEOL JEM-2100F equipped with a Schottky field emission gun and Gatan image filter and spectrometer (GIF Quantum 963SE). *In situ* X-ray diffraction (XRD) measurements were conducted on a PANalytical X'Pert Pro MPD equipped with a TTK-450 X-ray reaction chamber (Anton-Paar) while purging CO_2_ gas into the chamber. For the XRD measurements, an X-ray beam was generated at 45 kV/40 mA, and the generated X-ray beam wavelength was *λ* = 1.5418 Å (CuKα radiation).

### Corrosion evaluation

The initial corrosion assessment was carried out using H_2_ gas evolution measurements during immersion in 3.5 wt% NaCl (aq) at ambient temperature (22–23.5 °C). Untreated and CO_2_-treated AZ91D samples with 25 × 25 mm^2^ in size were prepared for the measurement as described in the following sentences. All samples were masked with a polymer tape to expose 0.833 cm^2^ circular area (10.3 mm diameter) to NaCl solution. Each H_2_ evolution measurement had one treated or untreated Mg alloy sample that was positioned upright in a plastic container containing 3000 mL of 3.5 wt% NaCl (aq) open to air. During the experiments, deionized water was added to the NaCl solution every 48 or 72 h to compensate for water evaporation. An inverted funnel filled with 3.5 wt% NaCl (aq) was placed over the tape-masked sample to collect H_2_ evolved during corrosion over the immersion time. After exposure, the samples were removed from the solution, rinsed with deionized water and then ethanol, dried using compressed air, and photographed for collection of graphical data.

Electrochemical impedance spectroscopy (EIS) and polarization measurements were also conducted for untreated (600 grit SiC finished) or treated AZ91D samples as working electrodes, with the exposed area of 0.833 cm^2^ (10.3 mm diameter) prepared by insulation tape masking. A reference saturated calomel electrode (SCE) and Pt plates with 3–5 cm^2^ of surface area for counter electrodes were used in 3.5 wt% NaCl solution open to air at ambient temperature. Before EIS or polarization measurement, an open-circuit potential (OCP) delay for 60 min was applied for the tape-masked AZ91D samples. EIS measurements after OCP delay were performed using the following parameters: ±10 mV amplitude with respect to the last corrosion potential (in OCP delay) and frequency range of 200 kHz to 7 mHz. For the impedance data fitting, Zview software version 4.0g (Scribner, Southern Pines, NC, USA) was used for two equivalent circuit models described later. For polarization measurements, the scanning was initiated at −2.25 V_SCE_ and stepped up to −0.95 V_SCE_ at a rate of 1 mV s^−1^.

### Tribology test

Dry sliding reciprocating wear tests using a tabletop Anton Paar pin-on-disk tribometer were employed to characterize the friction and wear resistance of AZ91D specimens. Tests were performed in compliance with ASTM G-133 (ASTM G-133 2022). A 6 mm-diameter hard silicon nitride (Si_3_N_4_) is used as a counter face to mitigate the ball wear during sliding. Tests were conducted in ambient air under a normal load of 1 N at a sliding frequency of 5 Hz. Wear tests were run for a total sliding cycle of 5000 (at 5 Hz) with a 6 mm stroke length corresponding to 120 m of sliding distance to study the steady-state friction behavior. Tribo 1.4x software was used to record the coefficient of friction (COF) and the wear tracks were analyzed using a Keyence white light interferometry (WLI). 3D profile of the wear tracks was measured and analyzed to calculate the wear volume loss (mm^3^) using VR-5000 series software. Wear rates (units of mm^3^ N^−1^ m^−1^) were calculated by dividing wear volume loss by normal load and the sliding distance. At least three tests were carried out at room temperature and the average value was reported.

## Results and discussion

### Initial characterization of CO_2_-treated Mg alloy surface

**Fig. 3:**
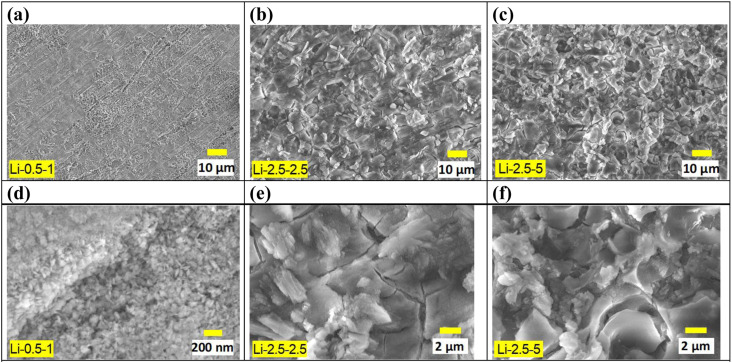
Microscopic surface morphonology changes of CO_2_ treated AZ91D over various pretreatment (NaHCO_3_ concentration) and salt loading amounts. (a and d) Li-0.5-1.0, (b and e) Li-2.5-2.5 and (c and f) Li-2.5-5.0.

Significant changes in surface morphology were observed after CO_2_ treatment on the Li salt-coated MgO surfaces. Initially, a pre-treated AZ91D sample, prior to CO_2_ exposure, shows separated islands of MgO/Mg(OH)_2_ layers, as presented in Fig. 2. This morphology can be explained by a low Pilling–Bedworth ratio (PBR) of MgO and Mg, reported to be 0.81, indicating that the oxide layer was fractured on the Mg substrate and unable to effectively suppress further oxidation and the inward diffusion of oxygen, thus lacking protective effects.^51^ Note that the observed cracks and voids may appear more pronounced under an SEM observation environment characterized by vacuum and near-zero humidity. Fig. 3 shows that CO_2_ treatment of AZ91D with pre-loaded Li salt transformed the separated island structure of the pre-treated MgO/Mg(OH)_2_ (Fig. 2) into a more connected layer with embedded crystalline particles on the surface. The size of crystals on the CO_2_-treated surfaces was smaller in the Li-0.5-1 condition than the others. When MgO/MgOH_2_ formed AZ91D was CO_2_ treated without Li salt, the surface morphology characterized as the separated island structure was maintained, as presented in Fig. S1 in the ESI,† which indicates that molten Li salt during thermal CO_2_ treatment accounts for the morphology change of the surface layer on AZ91D.

### Carbonate layer formation

**Fig. 4:**
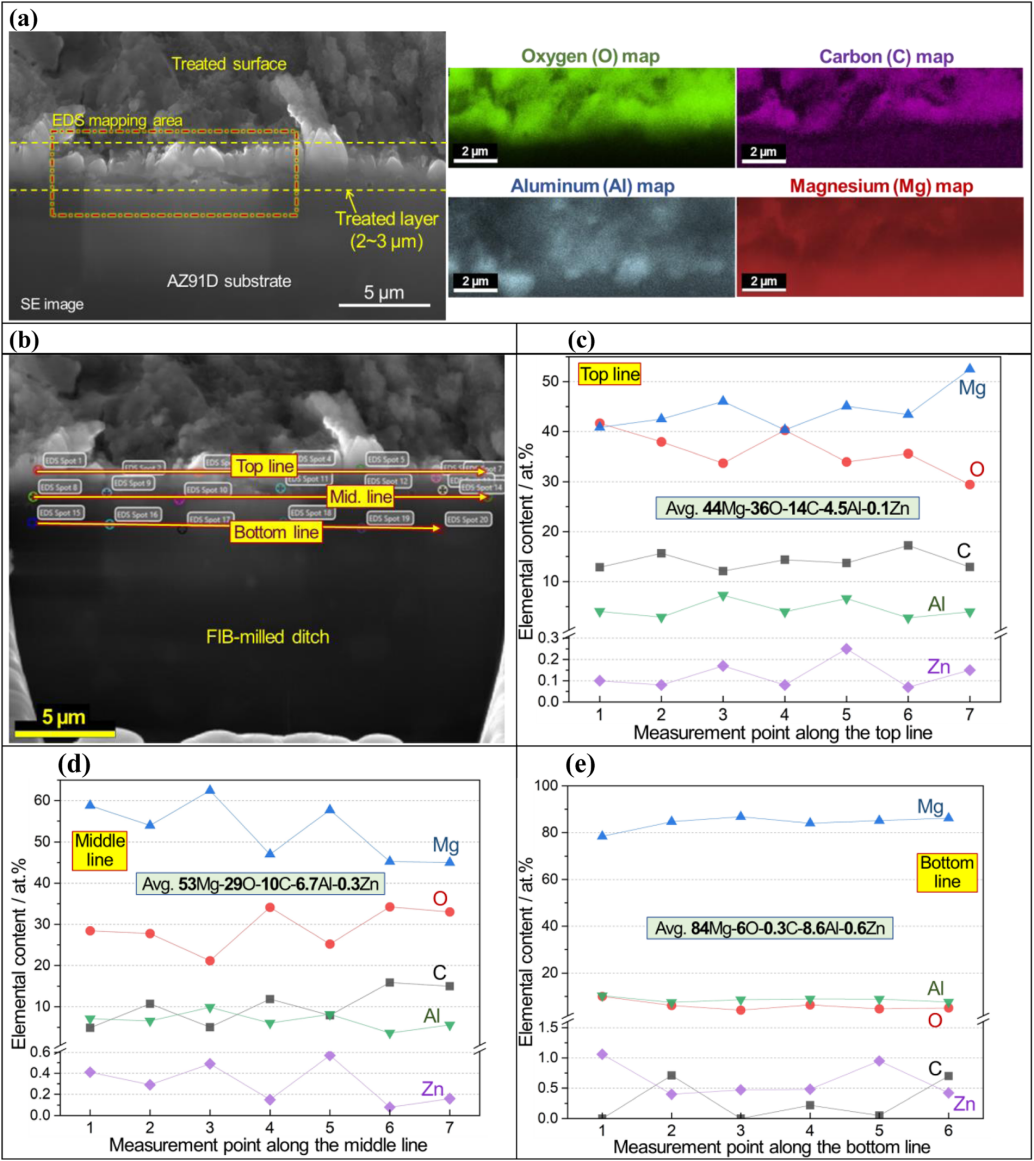
SEM-energy-dispersive X-ray spectroscopy (EDS) images of the CO_2_-treated AZ91D (*i.e.*, Li-0.5-1.0). (a) 45-degree tilted SEM image with corresponding elemental maps, (b) zoom-out of 45-degree tilted SEM images with point mappings across the layer, (c–e) Mg, O, C, Al and Zn composition profiles of the top, middle and bottom lines, respectively, along the layer in atomic percent.

An SEM image and EDS maps of CO_2_-treated AZ91D specimen (Li-0.5-1 condition) are presented in Fig. 4a, which revealed 2–3 micron thick surface layer containing Mg, O and C with locally-enriched Al. In Fig. 4b, point EDS measurement spots in the cross-sectioned sample (prepared by FIB milling) are presented. Notably, the top and middle lines of the layer showed high carbon contents (average values of ∼14 and ∼10 at% for the top and middle sections, respectively), as presented in Fig. 4c and d, while the bottom layer near the AZ91D substrate showed only trace amounts of carbon (average of 0.3 at%), as found in Fig. 4e. XPS depth profiling on the same sample revealed similar results where the carbon content was 20 at% at the top surface and gradually decreased to ∼5 at% at a depth of 1.3 μm [see Fig. S3x-2 in the ESI†]. While the presence of Li in the surface layer is highly probable, detecting Li using generic SEM/STEM/EDS can be challenging due to the low energy X-ray associated with the low atomic number (*Z* = 3) of Li.^52^ Further analysis regarding Li detection will be discussed using EDS with a newly designed window-less detector.^53^

**Fig. 5:**
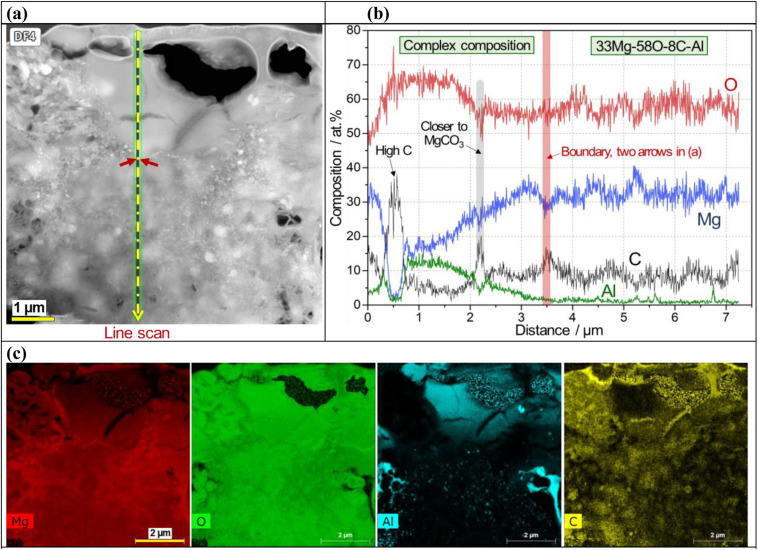
STEM/EDS analysis results presenting (a) cross-sectional medium angle ADF-STEM image of a CO_2_-treated AZ91D sample (Li-2.5-2.5), (b) a line EDS scan for Mg, O, Al and C profiles along the designated path in (a), and (c) spatial elemental mapping showing the distribution of Mg, O, Al, and C in the formed layer shown in (a).

The surface layer formed on the Li-2.5-2.5 AZ91D specimen was thicker than 7 μm, as confirmed by annular dark field (ADF) STEM with EDS characterization in Fig. 5 and S3 in the ESI.† A cross-sectional view of the layer, as shown in Fig. 5a, reveals at least 7 μm thick layer with void-like features on the upper region. EDS line scan of the CO_2_-treated layer in Fig. 5b exhibited complex composition profiles, including a high carbon spot, increased Al contents (3–14 at%) and probable MgCO_3_ phase in the top section but relatively flat Mg, O and C profiles (approximately 33Mg–58O–8C) in the lower section. In spatial EDS maps presented in Fig. 5c, Mg and O were uniformly distributed over the entire layer, while locally concentrated Al and C were also observed in the layer. As implied in the EDS line scan (Fig. 5b), C richer spots in the top portion of the layer (Fig. 5c) could be associated with the locations of MgCO_3_ crystalline phases.

**Fig. 6:**
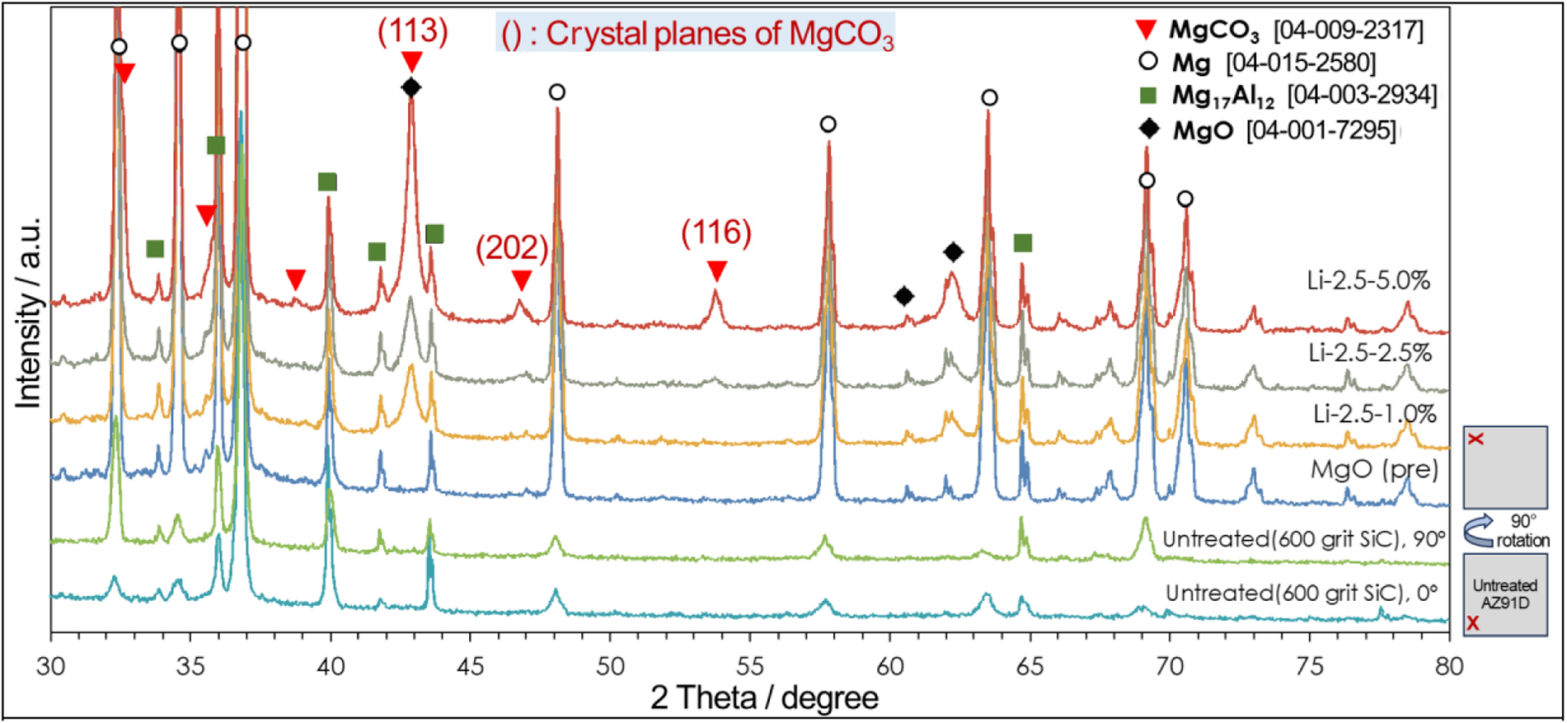
XRD patterns for untreated, MgO pre-treated and various thermal CO_2_-treated AZ91 specimens (with Li-salt loading conditions). XRD patterns of untreated Mg alloy were measured at two angles (0° and 90° configurations as described in the bottom right corner) to avoid the crystalline directional effect. For each phase, the international center for diffraction data numbers is presented in sqaure brackets.

XRD measurements, as presented in Fig. 6, confirmed the presence of MgCO_3_ in the resultant surface layer. Compared to an untreated AZ91D (with 600 grit SiC finish) and MgO pre-formed AZ91D, thermal CO_2_-treated AZ91D samples showed three distinctive intensity peaks at 43.0°, 46.5°, and 54.0°, which can be assigned to the (113), (202), and (116) planes of the hexagonal MgCO_3_ phase. For a treated specimen with a low Li concentration of 1.0% (Li-2.5-1.0), only one predominant peak at 43.0° was observed. However, as the Li salt concentration increased to 5.0%, two additional peaks at 46.5° and 54.0° emerged, likely due to the increased amounts of MgCO_3_ crystals in thicker surface layers. For example, the Li-0.5-1.0 specimen formed ∼3 μm thick layer (see Fig. 4a), whereas the Li-2.5-5.0 specimen formed a layer thicker than 7 μm (see Fig. 5).

The different layer thicknesses between Li-0.5-1.0 and Li-2.5-5.0 samples should have resulted from the different loadings of both NaHCO_3_ and LiNO_3_. While the effect of NaHCO_3_ may influence the initial MgO growth, the detailed growth kinetics is difficult to assess due to lack of data. However, the effect of LiNO_3_ loading on the layer thickness growth can be assessed by a previous study, which reported that the amount of CO_2_ absorbed in LiNO_3_ increased as the loading of LiNO_3_ increased to 20 mol% on MgO.^49^ By increasing the amount of CO_2_ in the molten salt, the reaction to form MgCO_3_ can be enhanced, so the layer thickness can be increased.

**Fig. 7:**
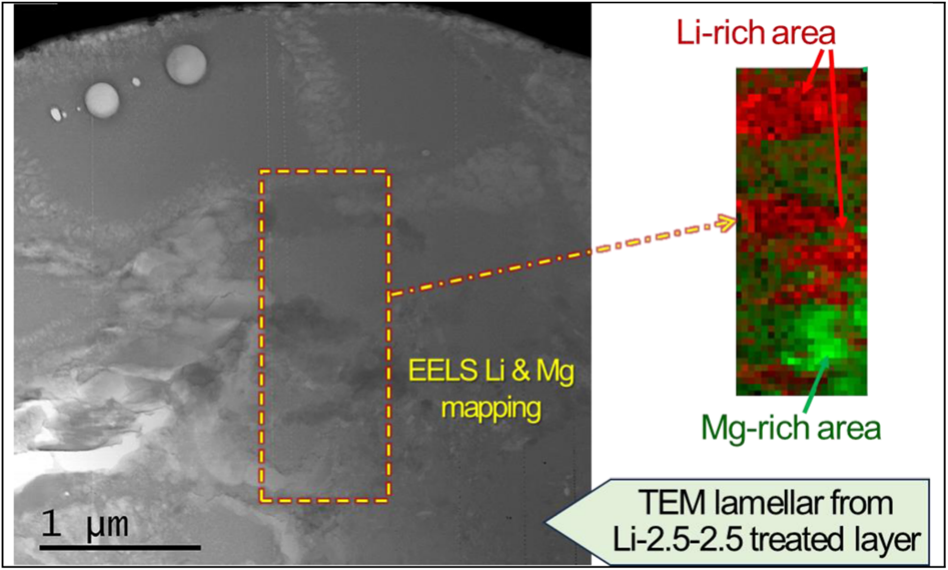
ADF-STEM survey image with colored EELS Li and Mg composite map in the Li-2.5-2.5 lamella.

The layer also contained a lithium compound. Lithium nitrate salt was used to facilitate the formation of MgCO_3_, but it also may have simultaneously reacted with CO_2_ to form Li_2_CO_3_. However, evidence of crystalline Li_2_CO_3_ was not initially found in the XRD and XPS analyses, as the post-cleaning procedure (*i.e.*, ultrasonication in deionized water for 5 min) must have washed off Li_2_CO_3_ on the top surface. Subsequent XRD and STEM characterization without washing the treated sample confirmed the presence of Li_2_CO_3_ crystals [Fig. S4 in the ESI†], which supports the formation of Li_2_CO_3_ from Li nitrate salt and CO_2_ reaction. To further characterize the presence of Li, an electron energy loss spectroscopy (EELS) survey in an ADF-STEM image was conducted, as presented in Fig. 7. The multiple linear least square fitting method was utilized to separate the overlapping Mg and Li edges at 51 eV and 55 eV, respectively, and Mg and Li richer areas in the surface layer are mapped using different colors. It is observed that Li richer area is associated with darker contrast in the ADF-STEM image.

### Layer formation mechanism

**Fig. 8:**
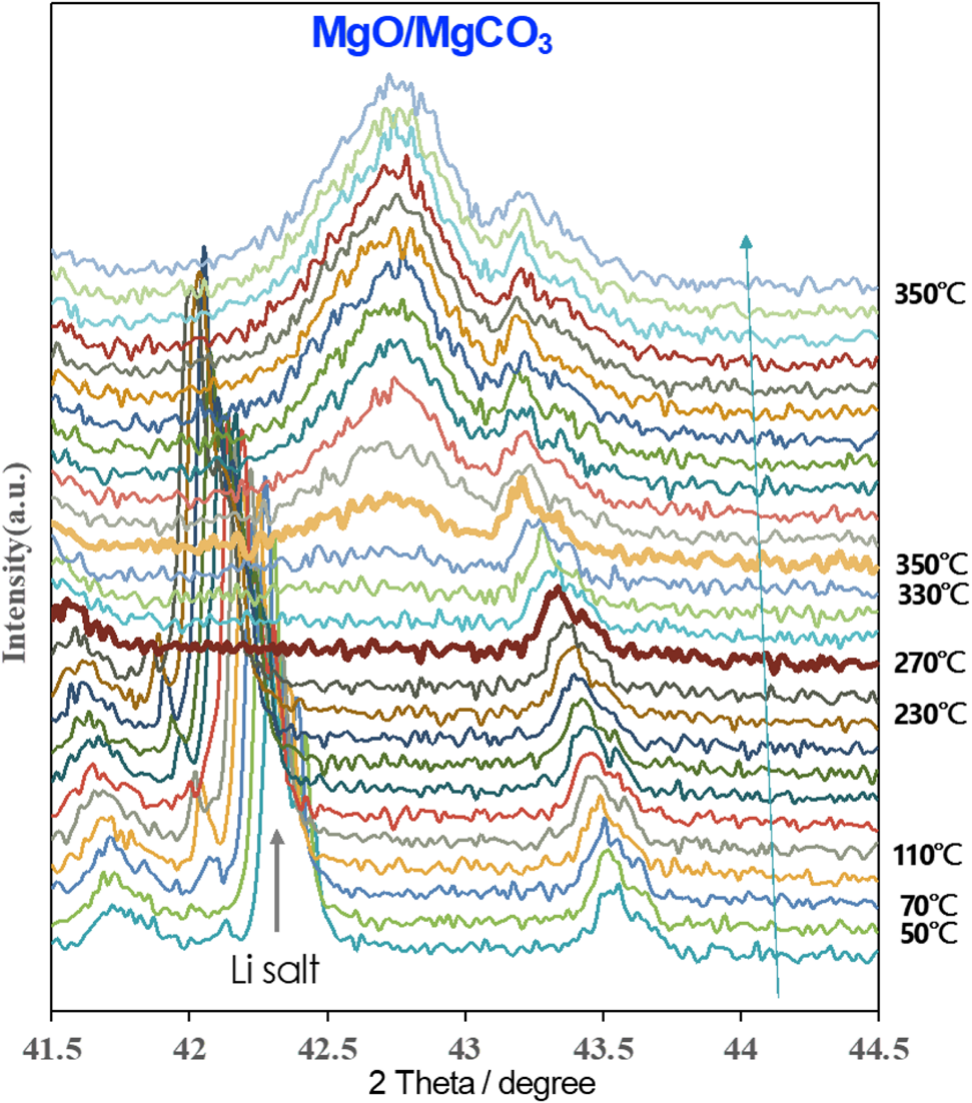
*In situ* XRD pattern measurement to understand MgCO_3_ crystalline growth. The assigned X-ray diffraction ranged from 41.5° to 44.5° and was scanned every 10 min while the temperature increased by 20 °C every 10 min under CO_2_ rich environment. When the temperature reached 350 °C, the isothermal condition was sustained for 3 h.

*In situ* XRD measurements were performed to understand the real-time formation of the carbonate layer through CO_2_ thermal treatment on Li salt-coated AZ91D, as shown in Fig. 8. During heating from room temperature to an intermediate temperature of 250 °C, a peak at 42.3° assigned to [113] of LiNO_3_ gradually shifted to smaller angles due to lattice expansion. The crystalline peak disappeared when the temperature reached 270 °C, corresponding to the melting point of LiNO_3_ at 255 °C. Subsequently, a new crystal peak at 42.7° associated with MgCO_3_ formation appeared at 330 °C, and the peak intensity (indicative of the volume fraction of crystals) increased over time. At 350 °C, the crystal growth reached a steady state after 90 min and showed no further change in XRD peaks until the end of CO_2_ treatment after 3 h. This real-time monitoring of XRD peak evolution provides insights into key reactions, such as Li salt melting, CO_2_ reactive absorption, and MgCO_3_ crystal-containing layer growth, as functions of temperature and time during the CO_2_ treatment.

### Corrosion mitigation evaluation by H_2_ collection measurement

**Fig. 9:**
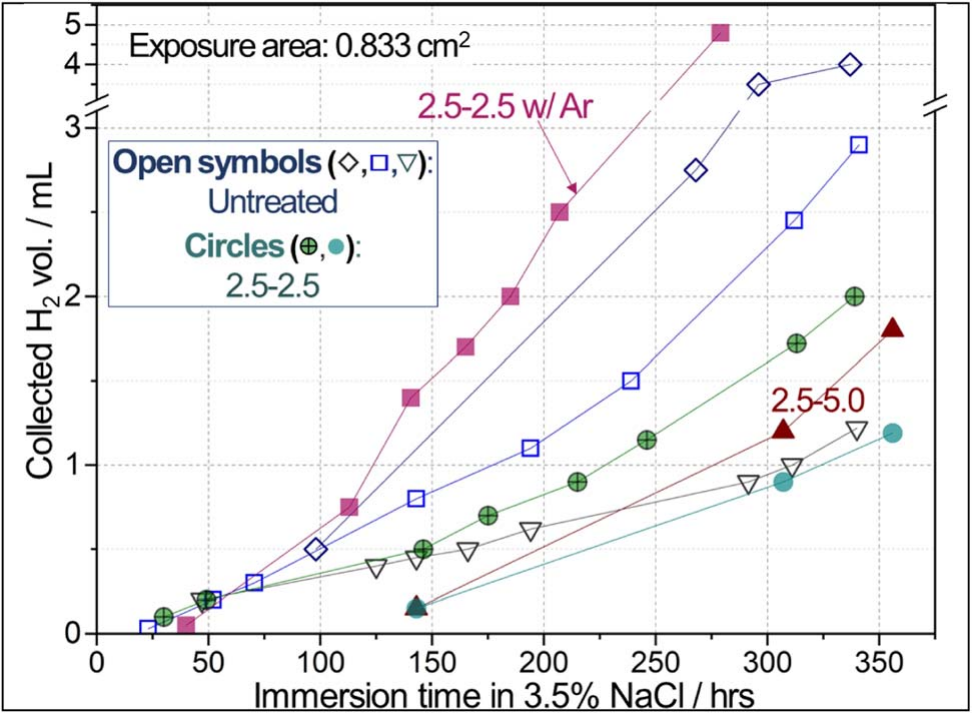
Hydrogen collection results of untreated and treated AZ91D samples (solid & patterned circles: Li-2.5-2.5, solid triangle: Li-2.5-5.0, solid squares: Li-2.5-2.5 w/Ar) compared to three untreated AZ91D samples (open symbols). Note that the sample immersion in 3.5% NaCl simulates a highly aggressive environment to Mg for accelerated evaluation of corrosion resistance.

The reconstructed surface exhibited a notable increase in corrosion resistance, as evidenced by preliminary H_2_ collection results. A photo of the H_2_ collection measurement setup is presented in Fig. S5a in the ESI.†Fig. 9 presents that the collected H_2_ volumes from the CO_2_-treated samples (*i.e.*, Li-2.5-2.5 and Li-2.5-5.0) were lower than those of the two untreated AZ91D samples for immersion times longer than 140 h in 3.5 wt% NaCl solution. An exception was that one untreated AZ91D showed low H_2_ volumes (downward triangle in Fig. 9). These notably different corrosion rates among the untreated AZ91D are presumably attributed to the local variations of cast microstructures. It was reported that the corrosion rates estimated by H_2_ collection data can vary up to a factor of 2.5 between the maximum and minimum data^54^ or ±44% deviation with respect to the average^55^ for AZ91D, as summarized in Table 3. It is also noted that AE42 and AM60 Mg alloys showed notable variations in H_2_ collection data. These previous results confirm that the relatively high variation of H_2_ volumes for the untreated AZ91D samples in Fig. 9 is a natural behavior.

**Table 3:** Summary of H_2_ collection literature data for different cast Mg alloys

Mg alloy	Corrosion exposure condition	Max. & min. H_2_ volume or converted data	Max./min.	Avg. H_2_ volume (*A*)	Standard deviation (*B*)	(*B*/*A*) × 100	Reference
AZ91D	5% NaCl for <100 h	∼5.5 & ∼3.5 mg per cm^2^ per day	1.57	—	—	—	Song 2001 (ref. 56)
AZ91D	0.1 M NaCl for 170 h	4 & 1.6 mL	2.5	—	—	—	Brady 2019 (ref. 54)
AZ31B	0.1 M NaCl for 170 h	24.2 & 18.9 mL	1.28	—	—	—	Brady 2019 (ref. 54)
AE42	3.5% NaCl for 168 h	1.2 & 0.77 mL cm^−2^	1.56	—	—	—	Dargusch 2021 (ref. 57)
AZ91D	0.6 M NaCl for 24 h	—	—	0.45 mL	±0.2 mL	±44%	Bland 2022 (ref. 55)
AM60	0.6 M NaCl for 24 h	—	—	0.3 mL	±0.3 mL	±100%	Bland 2022 (ref. 55)

**Fig. 10:**
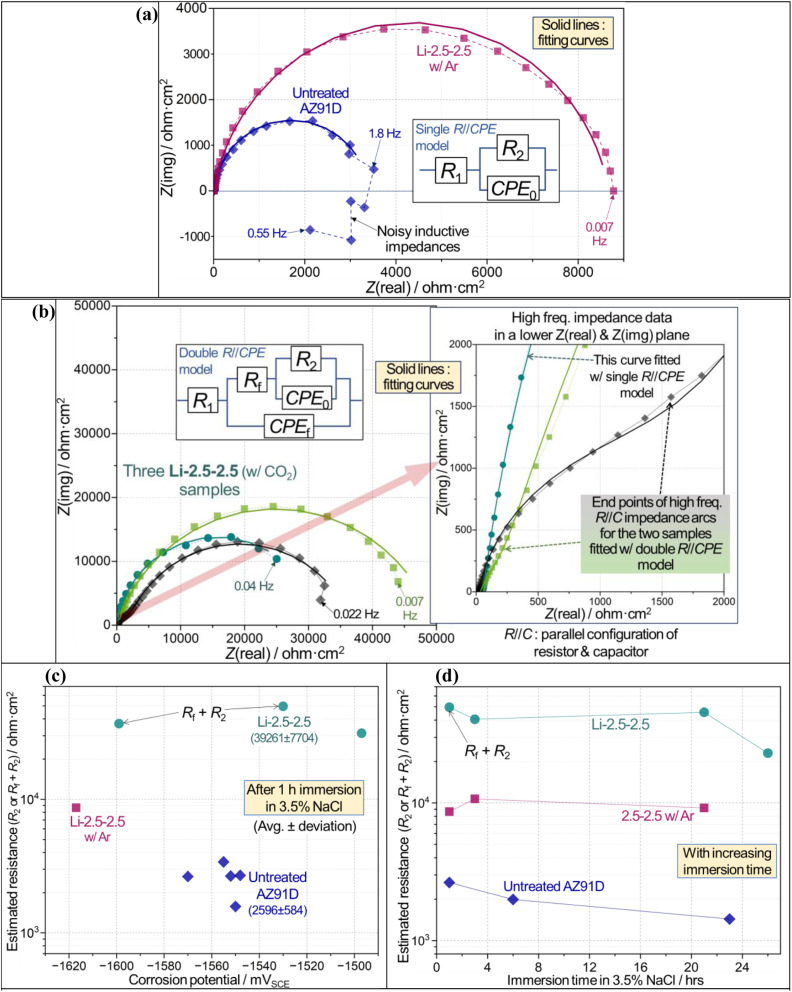
Electrochemical corrosion evaluation results for untreated (600 grit SiC finish) and Li-2.5-2.5 treated (with CO_2_ or Ar) AZ91D samples in 3.5 wt% NaCl solution. (a and b) Typical Nyquist impedance plots of untreated and treated AZ91D specimens with equivalent circuits as insets and data fitting curves. In (b), another inset on the right shows the impedance data in a lower impedance plane. *R*_2_ or *R*_f_ + *R*_2_ values (c) *versus* corrosion potential after 1 h immersion, and (d) with increasing immersion time. The detailed fitting results of impedance data are summarized in Tables S1–S3 in ESI.† The chi-square values of all fittings, reported in Tables S1–S3,† were lower than 0.002, which was used as a quality control for data fitting results.

**Fig. 11:**
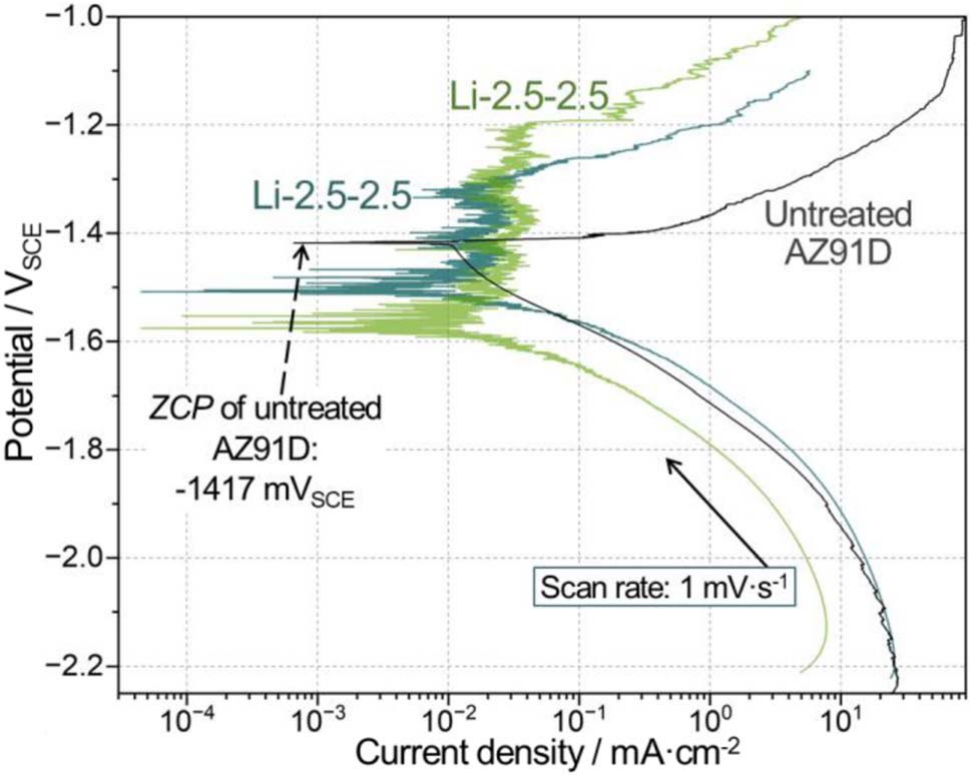
Comparison of polarization curves between one untreated and two individual Li-2.5-2.5 treated samples.

In visual inspection after the corrosion exposure, a CO_2_-treated AZ91D sample surface showed much smaller areas of corrosion attack compared to the untreated AZ91D [Fig. S5b and S5c in the ESI†]. The results of H_2_ collection are consistent with the electrochemical data discussed later in Fig. 10 and 11. In order to understand the effect of annealing without CO_2_ on the Li salt loaded sample (Li-2.5-2.5), thermal argon (Ar) treated samples (2.5-2.5 w/Ar) were prepared using the same process conditions. The Ar-annealed sample (filled square symbols in Fig. 9) exhibited higher H_2_ volumes than the untreated samples after 100 h, implying no corrosion mitigation effect.

### Electrochemical corrosion evaluation

CO_2_-treated AZ91D surface significantly increased corrosion reaction resistance, as revealed by comparison of impedance scales in Fig. 10a and b for both treated and untreated specimens in 3.5 wt% NaCl solutions for 1 h immersion. The scale (or size) of impedance was greater for the Li-2.5-2.5 CO_2_-treated specimen. It is also noted that two Li-2.5-2.5 treated AZ91D samples exhibit smaller impedance arcs, associated with *R*//*C* (parallel configuration of resistor and capacitor) behavior at high-frequency regime, before the larger major impedance arcs as seen in the right inset of Fig. 10b. To account for the different shapes of impedance spectra, two equivalent circuit models depicted in Fig. 10a and b were used for impedance data fitting, which would also enable quantitative evaluation of corrosion resistance in each untreated or treated AZ91D sample.

In the single *R*//CPE model (CPE: constant phase element) with *R*_2_ and CPE_0_, *R*_2_ is the charge transfer resistance of anodically dissolving metal(s) at the corroding interface, while CPE_0_ represents non-ideal capacitive behavior associated with the anodic metal dissolution. In the double *R*//CPE model with *R*_f_, *R*_2_, CPE_0_ and CPE_f_, *R*_f_ and CPE_f_ correspond to the resistance and non-ideal capacitance of permeable solid film, while *R*_2_ and CPE_0_ are the elements as described for the single *R*//CPE model. For both models, *R*_1_ is the solution resistance. The corrosion resistance of each sample was evaluated using *R*_2_ and *R*_f_ + *R*_2_ for the single and double *R*//CPE models, respectively, as reported previously.^50,58^ Note that there were other literature studies that took low-frequency inductive behavior into consideration for the corrosion evaluation of Mg and Mg alloys.^26,55,59,60^ However, a simpler impedance data fitting using the single *R*//CPE model without an inductive element is adopted here for untreated AZ91D, where inductive impedance was evident between 1.8 and 0.55 Hz (see Fig. 10a).

Fig. 10c indicates that an optimized CO_2_-treated sample (*i.e.*, Li-2.5-2.5) exhibits approximately 15 times greater corrosion resistance than the untreated surface (avg. resistances: 39 261 *vs.* 2596 ohm cm^−2^). This high corrosion resistance persisted for up to 26 h, as presented in Fig. 10d. Additionally, it appears that the inert Ar treatment results in an effective increase in impedance for a relatively short time immersion (see a square symbol in Fig. 10c and d), even though the formed layer was peeled off after washing and did not show corrosion protection in long-term H_2_ collection results (see Fig. 9).

Polarization measurements in Fig. 11 showed that CO_2_-treated AZ91D surfaces exhibited passivity-like behavior (between −1.55 and −1.2 V_SCE_), where Mg anodic dissolution was much smaller than the untreated AZ91D. While different zero current potential (ZCP) values were noted for the treated and untreated AZ91D samples, as seen in Fig. 11, no further discussion was made because ZCP values could fluctuate during dynamics polarization measurements and significantly differ from corrosion potentials. For example, the ZCP of untreated AZ91D (−1417 mV_SCE_, as indicated in Fig. 11) is notably more noble than the corrosion potential values (<−1540 mV_SCE_ as seen in Fig. 10c) during OCP measurements.

Considering the long-term H_2_ collection and electrochemical evaluation data, it is reasonable to state that CO_2_-treated AZ91D surfaces are distinctively more corrosion-resistant. The protection of Mg–C–O–Li and MgCO_3_ containing surface layer for AZ91D substrate is presumably attributed to a physical barrier effect limiting the permeation of corrosive species. Chemically induced corrosion mitigation, from the evolution of corrosion inhibitor(s) during the exposure, is open for further investigation.

### Wear property of the layer

**Fig. 12:**
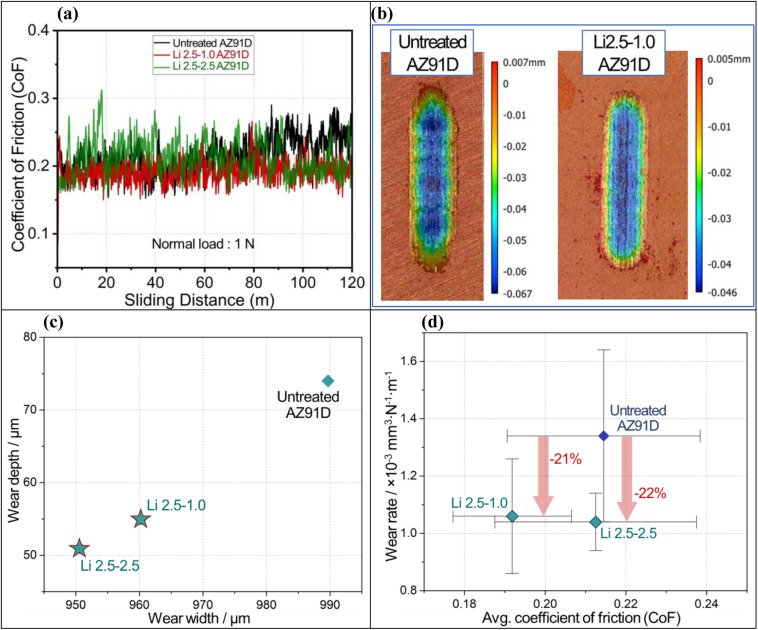
(a) Coefficient of Friction (CoF) *versus* sliding distance curves for all samples with a sliding distance of 120 m, (b) representative 3D surface profiles of the wear tracks on untreated and Li-2.5-1.0 treated AZ91D alloy samples showing the wear dimensions after 120 m of sliding, (c) 2D plot of the wear width and wear depth of all samples that were estimated from the 3D surface profiles, and (d) average CoF *versus* wear rate from the untreated and treated AZ91D alloy samples.

CO_2_-treated surface layers exhibited higher wear resistance, resulting in a lower wear rate. Typical coefficient of friction (CoF) curves as a function of sliding distance for untreated and CO_2_-treated AZ91D samples are plotted in Fig. 12a. The steady-state friction coefficients were obtained by dividing the mean friction force recorded during each experiment (after running-in) by the applied normal force. It is observed that all samples exhibited steady-state CoF in less than 3 meters (run-in period) of the total sliding distance, and there is no significant indication of fluctuations in the CoF over the entire sliding distance. The steady-state friction (*μ*_ss_ = 0.21 ± 0.02) amplitude for all the above conditions, despite the factor of a barrier layer, can be attributed to less plastic deformation of the worn surface and relatively low adhesion of the contact surfaces sliding at 1 N of normal load.

Fig. 12b illustrates comparative 3D wear surface maps of untreated and Li-2.5-1.0 AZ91D specimens as examples of raw wear data. The micro grooving, parallel to the sliding direction, visible on the wear tracks can be indicative of abrasive wear as the dominant wear mechanism. The depth and width of wear tracks for each AZ91D sample are plotted in Fig. 12c. It is evident that the wear dimensions of CO_2_-treated AZ91D samples, Li-2.5-2.5 and Li-2.5-1.0, were smaller than the untreated AZ91D specimen. This can be attributed to the resistance to plastic deformation when compared to that of the untreated sample. The average CoF and wear rate with the standard deviations are plotted in Fig. 12d. All CO_2_-treated AZ91D sample data indicate greater wear resistance compared to the untreated AZ91D. The average CoF was lower in the Li-2.5-1.0 sample (∼10% reduction) than the Li-2.5-2.5 and untreated AZ91D samples (as seen on the *x*-axis of Fig. 12d).

Interestingly, among the CO_2_ treated specimens, Li-2.5-2.5 AZ91D shows the lowest wear rate (1.04 × 10^−3^ mm^3^ N^−1^ m^−1^), which is ∼22% lower than that of the untreated AZ91D (1.34 × 10^−3^ mm^3^ N^−1^ m^−1^). The higher wear rate of untreated AZ91D can be attributed to the initial formation of discontinuous MgO upon sliding and then later dislodging of the oxides, causing severe abrasive wear on the exposed Mg metal. In contrast, the MgCO_3_ contatning barrier layer present on Li-2.5-2.5 AZ91 showed the lowest wear rate, which can be due to the partial smearing of the barrier layer in the matrix and increasing the shear strength of the substrate.^61^

## Conclusion

In this work, we have demonstrated that Li-salt-assisted thermal CO_2_ treatment on AZ91D resulted in the formation of a highly corrosion-resistant surface layer through CO_2_ absorption into the MgO layer with molten Li salt. High-resolution microscopic/chemical analysis and *in situ* XRD measurements were conducted to quantify the chemical composition of the barrier surface layer and elucidate the surface layer characteristics, including the presence of MgCO_3_ crystalline phase and uniform distribution of Mg, C and O, and the temperature (330 °C) associated with MgCO_3_ phase formation under molten Li salt and CO_2_ environment.

The corrosion protection and wear resistance performance were evaluated through electrochemical measurement, hydrogen collection and tribological characterization on various specimens. The CO_2_-treated AZ91D exhibited a 15-fold increase in long-term corrosion resistance (as assessed using impedance data) in 3.5 wt% NaCl and 21–22% lower wear rate (as revealed using wear volume data) compared to the untreated AZ91D specimen. Characterization results from SEM/STEM/EDS, STEM-EELS and XRD indicated that the unprotective MgO layer with separated island structure was completely transformed into a more compact and protective Mg–O–C–Li layer with MgCO_3_ crystalline phases.

The formation of a protective surface film, as demonstrated in this work, holds promise for environmentally friendly corrosion protection and excessive CO_2_ utilization. It opens new possibilities for protecting various existing Mg-based alloys for diverse applications. However, further optimization of the Li-salt-assisted CO_2_ thermal process is required for its application in complex Mg alloy structures. Each operational condition should be explored for various Mg alloy types with different thermal–mechanical properties and chemical reactivity to ensure effective protection.

## Conflicts of interest

There are no conflicts to declare.

## Supplementary Material

RA-014-D4RA02829E-s001
