# Supplementary material for: Lithium nitrate salt-assisted CO2 absorption for the formation of corrosion barrier layer on AZ91D magnesium alloy
Source: RSC Adv. 2024 Jun 3;14(25):17696–709. doi: 10.1039/d4ra02829e (PMC11145626; doi:10.1039/d4ra02829e)
Supplement: RA-014-D4RA02829E-s001 [file RA-014-D4RA02829E-s001.pdf]

Electronic Supplementary Material (ESI) for RSC Advances.
This journal is © The Royal Society of Chemistry 2024

Supporting information

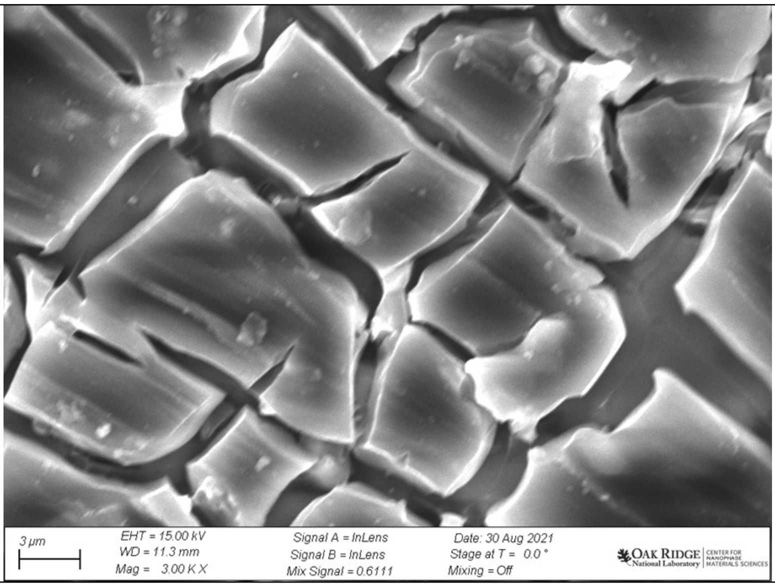

Figure S1. Preformed MgO AZ91D surface after CO_2 annealing at 350°C for 3 h without Li salt loading.

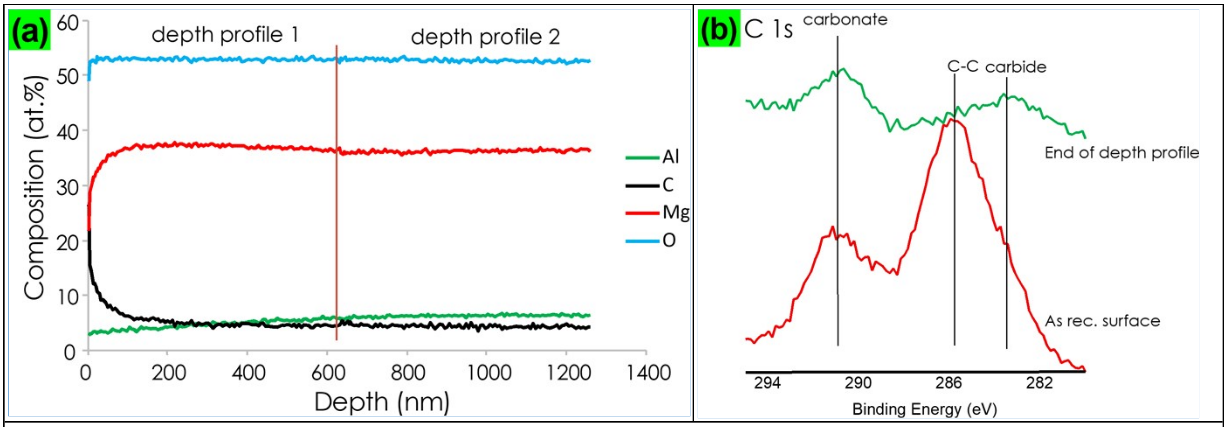

Figure S2. (a) XPS depth profiling of CO_2 annealed sample (Li-0.5-1.0) and (b) XPS pattern of C 1s for before and after depth profile.

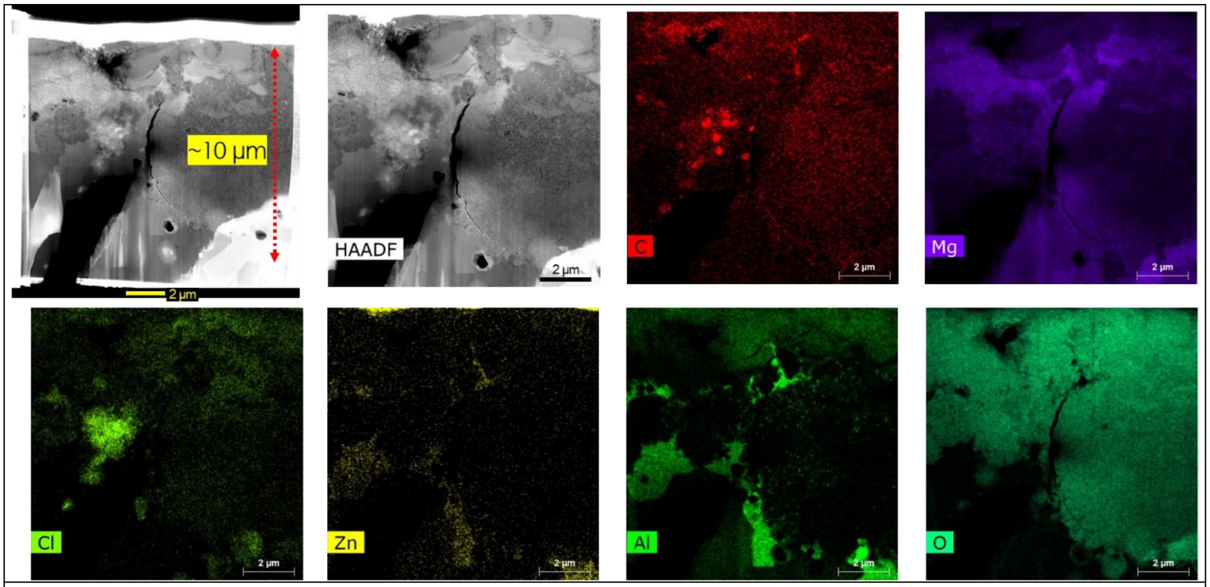

Figure S3. High-magnification cross-sectional (high angle ADF-STEM) images of the CO_2-annealed AZ91D sample (Li-2.5-2.5) along with corresponding energy-dispersive X-ray spectroscopy elemental mapping showing the distribution of Mg, O, Al, C, Cl and Zn in the formed layer (10 µm)

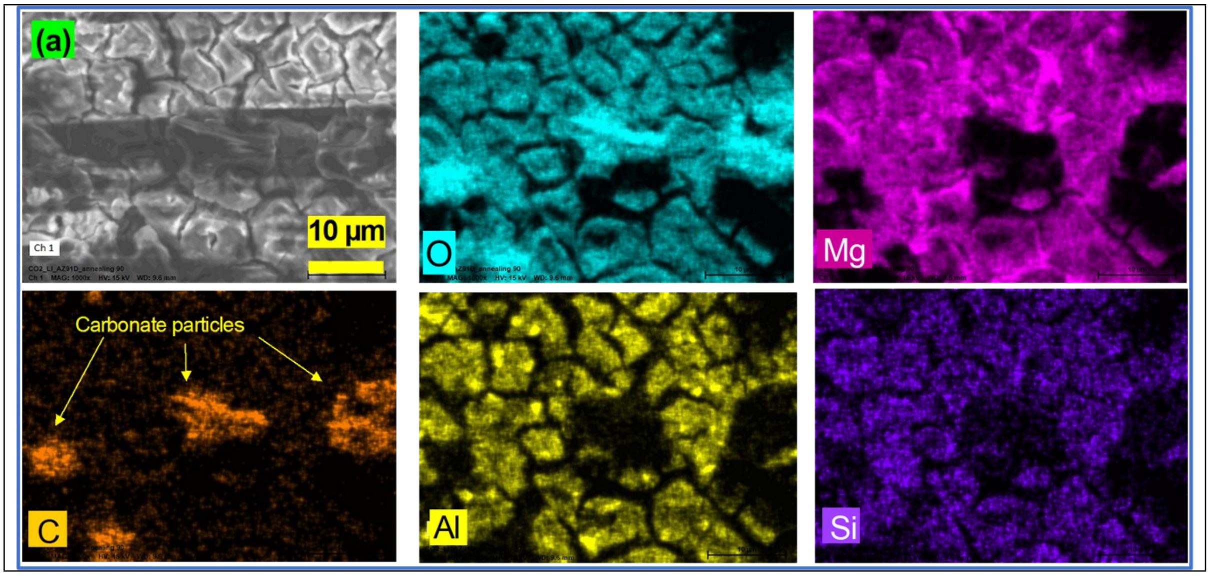

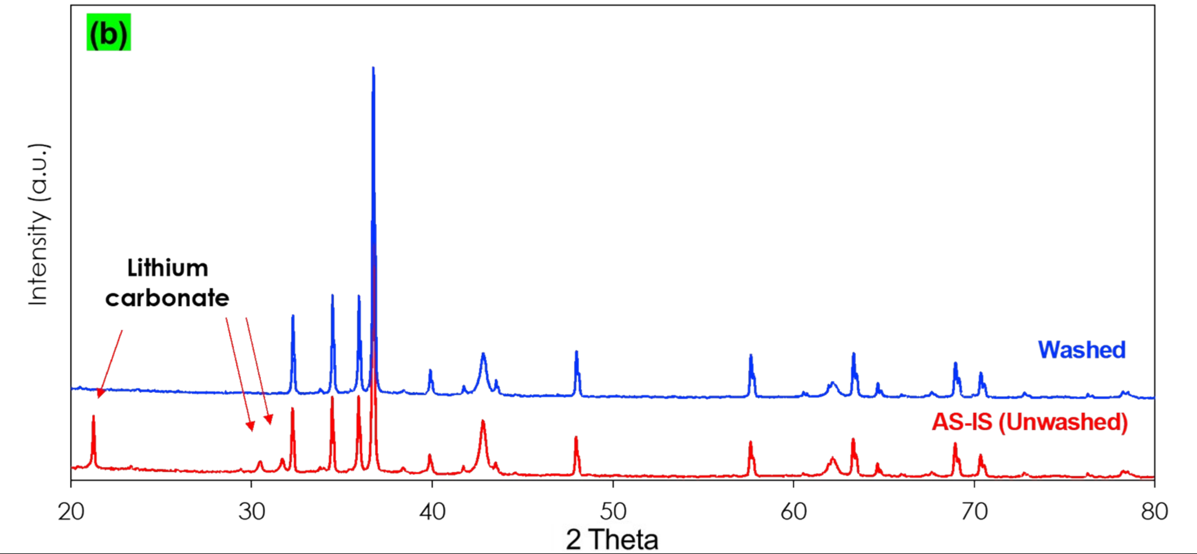

Figure S4. (a) SEM-EDS of CO_2-annealed AZ91D (i.e., Li-2.5-2.5) without washing (b) corresponding XRD measurements of as prepared vs. washed samples.

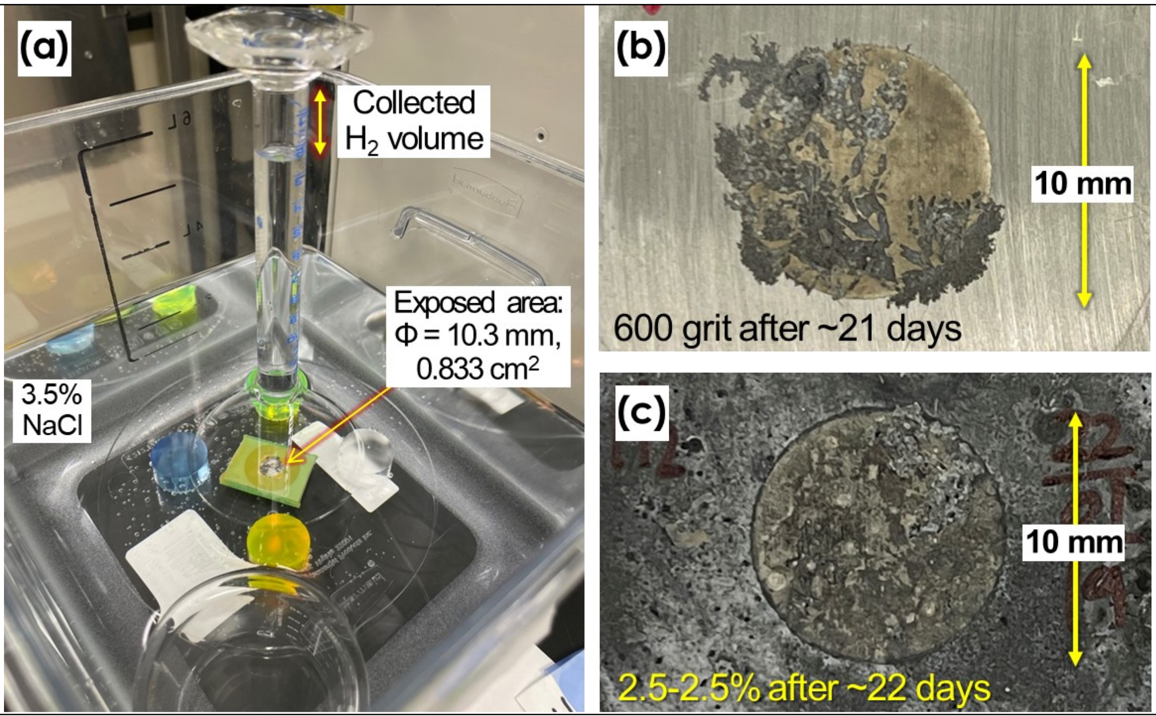

Figure S5. Corrosion mitigation effects of CO_2-treated layer on the Mg specimen: photo-images of the untreated 600 grit-finished AZ91D (a) and CO_2-treated AZ91D (b) specimens (exposed surface area: 0.833 cm^2) after 21 and 22 day immersion in 3.5 wt.% NaCl solution. (c) Hydrogen collection test of the CO_2-treated samples compared with an untreated sample.

Table S1. Summary of impedance data fitting for untreated AZ91D with short- and long-term immersion.

Sample ID	Short-term immersion (4 individual samples)				Untreated AZ91D long-term measurement (1 sample with 3 measurements)
	u1	u2	u3	u4	

Immersion time		1 h	1 h	1 h	1 h	1 h	6 h	23 h
Corrosion potential / mV_{SCE}		-1552	-1550	-1548	-1555	-1570	-1572	-1564
R_1 / ohm		10	7.8	13.4	6.2	14	14	16
R_2 / ohm·cm^2		2665	1576	2697	3400	2644	1991	1434
CPE_0	Capacitance / F·cm^{-2}	1.06E-5	8.81E-6	1.07E-5	6.7E-6	1.07E-5	1.3E-5	1.76E-5
	n	0.94	0.95	0.93	0.94	0.93	0.91	0.9
Chi-squared		1.6E-3	1.9E-3	1.6E-3	1.2E-3	8.7E-4	1.2E-3	1.3E-3

Table S2. Summary of impedance data fitting for Li2.5-2.5 with Ar treated AZ91D with long-term immersion to 21 h.

Sample ID		Li 2.5-2.5 w/ Ar AZ91D long-term measurement (1 sample with 3 measurements)		
Immersion time		1 h	3 h	21 h
Corrosion potential / mV_{SCE}		-1617	-1589	-1574
R_1 / ohm		8.6	9.2	16.8
R_2 / ohm·cm^2		8656	10719	9204
CPE_0	Capacitance / F·cm^{-2}	1.31E-4	1.15E-4	4.02E-5
	n	0.91	0.94	0.91
Chi-squared		1.3E-3	1.6E-3	1.9E-3

Table S3. Summary of impedance data fitting for Li2.5-2.5 treated AZ91D with short- and long-term immersion.

Sample ID		Short-term immersion (2 individual samples)		Li 2.5-2.5 AZ91D long-term measurement (1 sample with 4 measurements)			
		t1	t2				
Immersion time		1 h	1 h	1 h	3 h	21 h	26 h
OCP / mV_{SCE}		-1497	-1559	-1530	-1515	-1498	-1514
R_1 / ohm		67	24	40	41	65	66
R_2 & R_f / ohm		37552 & -	41142 & 3069	58384 & 1250	48757	54754	27649
R_2 / ohm·cm^2		31281	-	-	40615	45610	23032
$R_f + R_2$ / ohm·cm^2		-	36828	49675	-	-	-
CPE_0	Capacitance / F·cm^{-2}	6E-5	5.37E-6	3.78E-5	5.87E-5	6.57E-5	6.15E-5
	n	0.92	0.88	0.79	0.83	0.91	0.92
CPE_f	Capacitance / F·cm^{-2}	-	2.99E-5	1.36E-5	-	-	-
	n	-	0.8	0.9	-	-	-
Chi-squared		5.2E-4	4.6E-4	4.3E-4	1.9E-3	1.8E-3	1.8E-3
